# Subglottotracheal Adenoid Cystic Carcinoma in a 16-Year-Old Female—A Case Report

**DOI:** 10.3390/medicina59061140

**Published:** 2023-06-13

**Authors:** Cristina Ștefania Dumitru, Nicolae Constantin Balica

**Affiliations:** 1 Department of Microscopic Morphology/Histology, Angiogenesis Research Center, “Victor Babes” University of Medicine and Pharmacy, Sq. EftimieMurgu No. 2, 300041 Timisoara, Romania; 2 Department of Ear-Nose-Throat, “Victor Babes” University of Medicine and Pharmacy, Eftimie Murgu Square 2, 300041 Timisoara, Romania; balica@umft.ro

**Keywords:** adenoid cystic carcinoma, subglottotracheal, pediatric patient, management

## Abstract

Cystic adenoid carcinoma (ACC) is a rare malignant epithelial tumor arising from exocrine glands and accounts for only 1% of head and neck cancers. ACCs are common in the fifth and sixth decades of life, predominantly in women, and characterized by slow progression, local aggression, recurrence, and high metastasis. Subglottotracheal ACC is a rare tumor in the pediatric population, with only a few cases reported in the literature. We present a case of a 16-year-old female who was diagnosed with ACC in the subglottic and tracheal region. The patient presented with respiratory failure but without a history of dysphonia, dyspnea, stridor, or dysphagia. The diagnosis was confirmed by a biopsy, and subsequent imaging studies showed a large tumor involving the subglottic and tracheal region. The therapeutic management of this patient has been challenging due to the rarity of this tumor in the pediatric population and the potential long-term complications associated with tumor recurrence and psychological impact. This case highlights the diagnostic and therapeutic challenges in the management of subglottotracheal ACC in children and the importance of a multidisciplinary approach to optimize patient outcomes.

## 1. Introduction

Head and neck carcinoma is the sixth most common cancer worldwide and accounts for approximately 4% of all cancer cases. The incidence of head and neck cancer varies depending on the specific type of cancer and the location [1]. Squamous cell carcinoma of the head and neck (HNSCC) is the most common type of head and neck cancer worldwide, accounting for more than 90% of all cases. Other types of head and neck cancer include salivary gland tumors, nasopharyngeal carcinoma, and sinonasal tumors. Adenoid cystic carcinoma (ACC) is a rare type of cancer that can occur in various parts of the body, including the head and neck region. ACC commonly arises from the salivary glands but can also occur in other areas such as the lacrimal gland, breast, and trachea [2].

ACC is characterized by the presence of specific molecular alterations that contribute to its clinical behavior. One of the most common molecular alterations is a chromosomal translocation, t (6;9) (q22–23; p23–24), which leads to the fusion of the MYB proto-oncogene with the NFIB gene (Nuclear Factor I B). This fusion gene is present in more than 50% of ACC cases and is considered a hallmark of the disease. Other less frequent genetic alterations include mutations in the PIK3CA, NOTCH1, and HRAS genes [3,4].

The MYB-NFIB fusion protein is thought to drive the development and progression of ACC by activating the expression of genes involved in cell proliferation, survival, and migration. The presence of this fusion gene has also been associated with a better prognosis and a lower risk of distant metastasis in some studies. In addition, other molecular pathways and alterations have been identified as playing a role in the development and progression of ACC, such as alterations in the p53 pathway, dysregulation of miRNAs, and alterations in the tumor microenvironment [5]. Understanding the clinical and molecular features of ACC is critical for its diagnosis and treatment. New therapies targeted at specific molecular alterations are being developed, which may improve outcomes for patients with ACC [6].

In the head and neck region, ACC typically presents as a slow-growing, painless mass that may compress surrounding tissues or invade nearby structures. It is often difficult to diagnose because it can mimic other benign or malignant lesions and requires a combination of imaging, biopsy, and molecular testing to confirm the diagnosis. The treatment of ACC depends on the location, size, and extent of the tumor, as well as the patient’s overall health and medical history. Surgery is the primary treatment option and involves the removal of the tumor and adjacent tissues, which may be followed by radiation therapy and/or chemotherapy. However, because ACC tends to be resistant to conventional treatments, patients may require ongoing monitoring and follow-up care [7].

The prognosis for ACC varies widely and depends on several factors, such as the location and stage of the cancer, the completeness of surgical resection, and the molecular characteristics of the tumor. Some patients may experience a recurrence of the cancer or develop distant metastases, which can be challenging to treat. Overall, ACC is considered a difficult cancer to manage and requires a multidisciplinary approach involving specialists in surgery, radiation oncology, medical oncology, and supportive care [8].

## 2. Materials and Methods

Detailed Case Description: A 16-year-old girl presented to the ORL (Otorhinolaryngology) Emergency Service with respiratory failure. The respiratory distress observed in the patient, characterized by tachypnea and use of accessory muscles, indicated significant difficulty in breathing. These symptoms were particularly evident during mild physical effort. It was reported that the patient’s oxygen saturation was less than 90% without oxygen therapy during minimal physical effort. The patient had no significant medical history, and there was no history of dysphonia, dysphagia, cough, or hoarseness. The only symptoms reported were progressive and persistent dyspnea over the last 5 days and mild biphasic stridor in the last 2 days.

**Figure 1:**
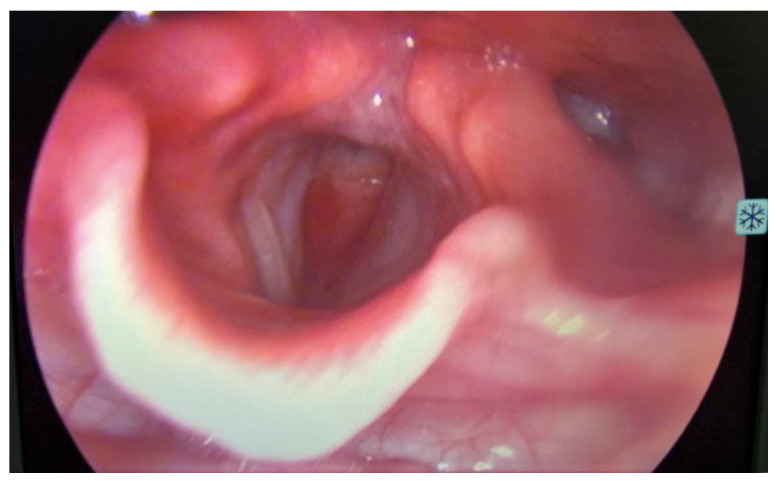
Laryngoscopy revealed a bulging subglottic mass, without visualization of the first tracheal ring, with marked narrowing of the respiratory space, normal appearing supraglottic and glottic area.

On ORL (Otorhinolaryngology) examination, the patient was in severe respiratory distress, with tachypnea, use of accessory muscles, and showing no lymph nodes or masses at visual inspection and palpation of the neck. At laryngoscopy (Figure 1), supraglottic areas showed a normal appearance, free vocal cords, with preserved mobility, and a subglottic tumor formation located at the level of the first tracheal ring, with marked narrowing of the respiratory space.

**Figure 2:**
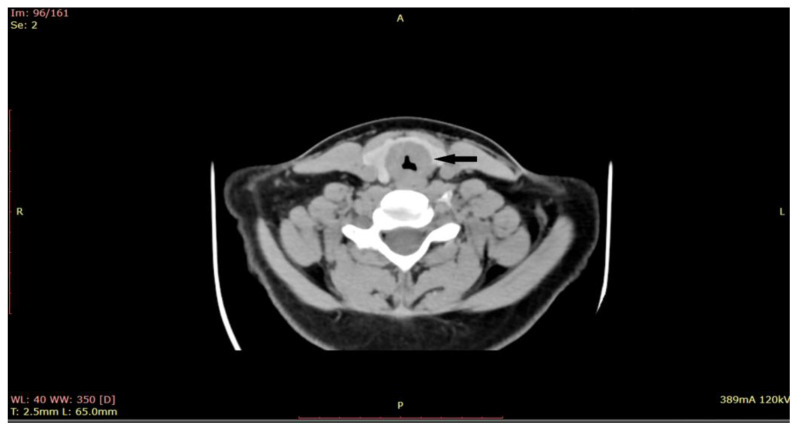
CT of the larynx shows a subglottic submucosal mass with marked intraluminal stenosis (at the arrow)-axial plane.

Given the critical nature of the patient’s condition, an immediate intervention was performed before sending the patient for a CT scan. Emergency tracheostomy was performed to secure the airway. However, during the operation, the patient developed a spontaneous left pneumothorax, which necessitated pleural drainage. The aim of the procedure was to secure the airway, relieve respiratory distress, and obtain a biopsy for further diagnosis. Once the patient’s condition stabilized after the pleural drainage, a biopsy of the subglottic tumor was obtained through direct laryngoscopy. During the direct laryngoscopy procedure, a tumor formation was observed in the subglottic region, involving the first tracheal ring. On examination, the tumor appeared slightly indurated, with smooth local mucosa. It circumferentially covered the subglottic area with marked narrowing of the respiratory space. A computed tomography (CT) scan was performed (Figure 2), which showed subglottic mass involving the trachea, with marked circumferential and slightly asymmetrical thickening of the laryngeal walls, from a plane passing through the arytenoid cartilages, involving the cervical trachea until the distal space C7-T1, with marked intraluminal stenosis (71%) and compression of the thyroid lobes.

Further investigations: Chest X-ray was normal, with no evidence of distant metastasis. Blood tests were normal, and oxygen saturations were less than 93% without oxygen therapy.

**Figure 3:**
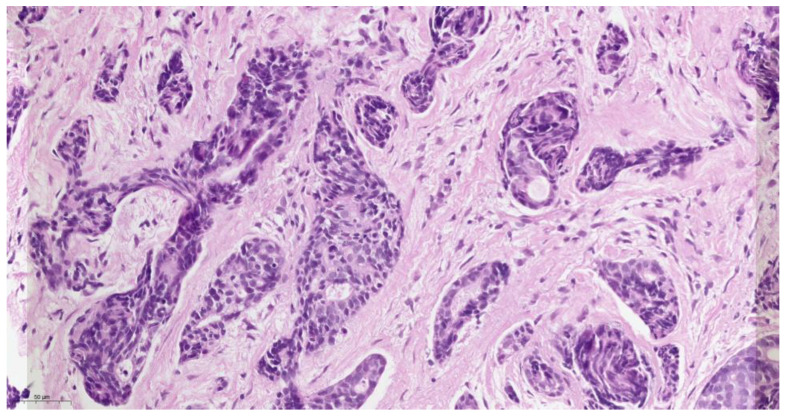
Hematoxylin-eosin staining of ACC, original magnification ×400.

Histopathological examination (Figure 3) of the biopsied tissue indicated the diagnosis of cystic adenoid carcinoma, with the presence of glandular, cribriform, and predominantly solid growth patterns. The tumor is composed of small, rounded cells with a small amount of cytoplasm, basophilic staining, and a large, rounded nucleus with a small nucleus. The stroma of the tumor is desmoplastic, which means that there is an overgrowth of fibro-conjunctive tissue around the tumor.

The management of a patient with subglottotracheal adenoid cystic carcinoma (ACC) usually involves a multidisciplinary approach. In this case, the patient’s case was presented at a multidisciplinary meeting (including otolaryngologists, radiation oncologists, pediatric medical oncologists, pathologists, radiologists, and pediatricians) where treatment options were discussed. Treatment options available to the patient included surgery (laryngectomy), radiotherapy, chemotherapy, and proton radiotherapy. According to the case report, the patient, along with her family, opted for proton radiotherapy at another medical center with extensive experience in pediatric oncology in head and neck cancers.

Six months after proton radiotherapy, the patient returned to our medical center in order to close the tracheal stoma. The patient has also been followed closely for the past two years with imaging studies and regular clinical examinations. To date, there has been no evidence of recurrence or metastasis, and the patient has had no significant long-term effects following treatment.

## 3. Discussion

Adenoid cystic carcinoma (ACC) is a rare malignancy with unique clinical and pathological features. The etiology of ACC in pediatric patients is not well understood. However, it has been suggested that exposure to environmental toxins and radiation therapy may play a role in the development of this cancer. While the exact pathogenesis of ACC remains incompletely understood, recent studies have shed light on several key molecular and genetic alterations that contribute to its development and progression. One important characteristic of ACC is its slow growth rate and high propensity for perineural invasion. Studies have suggested that this perineural invasion may be facilitated by the expression of specific proteins, such as S100 and nerve growth factor receptor (NGFR), in ACC cells [9]. ACC is also associated with specific genetic alterations, including chromosomal translocations involving the MYB or MYBL1 genes. These translocations result in the fusion of MYB or MYBL1 with other genes, leading to the expression of abnormal fusion proteins that may contribute to the development and progression of ACC [10]. In addition to genetic alterations, ACC is associated with dysregulation of several signaling pathways. For example, studies have shown that ACC cells have increased expression of Notch signaling components, which are involved in cell survival, proliferation, and differentiation. Other studies have suggested that the PI3K/AKT/mTOR signaling pathway is also dysregulated in ACC, contributing to tumor growth and survival [11,12]. Overall, the pathogenesis of ACC is complex and multifactorial, involving both genetic and molecular alterations. A better understanding of these mechanisms may lead to the development of targeted therapies for this challenging malignancy.

The histopathology of ACC is characterized by a biphasic growth pattern, with a combination of solid, cribriform, and tubular structures. The tumor cells have a characteristic “Swiss cheese” appearance due to the presence of pseudocysts formed by tumor cells. The pseudocysts are lined by epithelial cells that secrete mucinous or hyaline material. The tumor cells are typically small and uniform in size, with scant cytoplasm and a round to oval nucleus. ACC has a tendency to invade surrounding tissues and can infiltrate nerves, blood vessels, and bone [13]. In our case, histopathological grading was predominantly solid. This indicates that the tumor had a solid growth pattern, with less evidence of the typical cribriform and tubular structures that are commonly seen in ACC. The solid growth pattern of ACC was associated with a more aggressive clinical course and a higher risk of metastasis.

Subglottic and tracheal adenoid cystic carcinoma (ACC) in pediatric patients is a rare cancer that can be challenging to manage. The diagnosis of subglottotracheal ACC in pediatric patients can be challenging, as symptoms can be non-specific and similar to those of other respiratory pathologies. The symptoms may be related to the location of the tumor and the extent of involvement of surrounding structures. Some common symptoms of subglottic and tracheal ACC in pediatric patients include: respiratory symptoms (difficulty breathing, stridor, wheezing, coughing, and shortness of breath), voice changes (hoarseness, change in voice pitch, and vocal weakness are common symptoms of subglottic ACC), dysphagia (the tumor invades the esophagus), neck swelling (the involvement of the lymph nodes), and recurrent respiratory tract infections (the tumor may cause recurrent respiratory infections, which can be mistaken for asthma or bronchitis). It is important to note that the symptoms of subglottic and tracheal ACC in pediatric patients may be subtle and nonspecific. Therefore, a high index of suspicion is needed to diagnose the condition in a timely manner. An early diagnosis can lead to better treatment outcomes [14].

Therefore, clinical support, including imaging studies such as computed tomography (CT) or magnetic resonance imaging (MRI), and bronchoscopy are important tools in the diagnostic process. Treatment for subglottotracheal ACC in pediatric patients typically involves a multidisciplinary approach, including surgery, radiation therapy, and chemotherapy. The choice of treatment depends on several factors, including the location and size of the tumor, the extent of invasion, and the patient’s age and overall health status [15,16,17].

Surgery is usually the main treatment, with the goal of completely removing the tumor while preserving organ function and quality of life. In some cases, partial laryngectomy or tracheal resection may be necessary, which can result in long-term airway complications and the need for tracheostomy [18,19]. In the case of subglottic ACC, surgery may be combined with adjuvant radiation therapy to reduce the risk of local recurrence. However, in pediatric patients, radiation therapy is associated with a higher risk of long-term side effects, such as growth retardation, hormonal dysfunction, and cognitive impairment, and therefore should be used with caution and careful consideration of the potential risks and benefits [20].

Other treatment options for subglottotracheal ACC in pediatric patients include radiotherapy, chemotherapy, immunotherapy, and targeted therapy, but their effectiveness in this patient population is not well established, and they are typically reserved for cases of advanced or recurrent disease [21].

The use of radiotherapy and chemotherapy is controversial due to the potential long-term side effects on growth, development, and organ function. However, in some cases, they may be necessary to achieve optimal tumor control and improve survival. Radiotherapy has been shown to be effective in reducing the risk of local recurrence and improving overall survival in patients with ACC, but its use in pediatric patients is associated with a higher risk of long-term complications. Therefore, its use should be carefully considered, and the dose and duration of treatment should be tailored to the individual patient’s needs [22,23].

Chemotherapy has limited efficacy in the treatment of ACC, with low response rates and limited survival benefit reported in most studies. Its use may be considered in combination with radiotherapy or as a palliative treatment for advanced or recurrent cases. However, in pediatric ACC, there are limited data on the efficacy of chemotherapy, and studies suggest it does not improve survival outcomes compared to patients who do not receive chemotherapy. Furthermore, chemotherapy is associated with a higher rate of adverse events and does not prevent distant metastases [24,25].

Proton therapy has been used in the treatment of subglottic and tracheal ACC in pediatric patients with good outcomes. Proton therapy is a form of external beam radiation therapy that uses protons instead of X-rays to treat cancer. Protons deposit their energy directly in the tumor and then stop, reducing the amount of radiation exposure to healthy tissues surrounding the tumor. This can be particularly beneficial in the treatment of pediatric patients, where radiation exposure to healthy tissues can have long-term effects [26,27]. Proton therapy does have some limitations, including the need for specialized equipment and expertise, as well as the high cost. However, it may be a valuable treatment option for pediatric patients with subglottic and tracheal ACC, particularly when other treatments, such as surgery, are not feasible or not desired by the patient [28].

Proton therapy is a highly precise form of radiation therapy that can target tumors while sparing surrounding healthy tissues. This can be especially important in pediatric patients, who may be more susceptible to radiation-related side effects. In our case of the 16-year-old girl with subglottotracheal adenoid cystic carcinoma, proton therapy was chosen in another center as a treatment option. After 2 years of follow-up in our center, there were no observed side effects from the proton therapy. This is encouraging, as it suggests that proton therapy may be a viable treatment option for pediatric patients with head and neck cancers, including adenoid cystic carcinoma. The management of subglottotracheal ACC in pediatric patients also includes supportive care, such as nutritional support, pain management, and psychological support for the patient and their family. However, it is important to note that longer-term follow-up is needed to fully evaluate the efficacy and potential side effects of proton therapy in this patient.

As is well known, ACC is a slow growing but aggressive malignancy that has a high rate of recurrence, even after successful treatment. The risk of recurrence and metastasis increases with higher tumor stage, positive margins, and perineural invasion [29]. The prognosis for ACC depends on various factors, including the site and stage of the tumor, the histological subtype, and the presence of certain molecular markers. The 5-year survival rate for ACC ranges from 40% to 89%, depending on these factors. Patients with ACC require long-term follow-up and monitoring to detect any signs of recurrence or metastasis. Treatment options for recurrent or metastatic ACC include surgery, radiation therapy, chemotherapy, targeted therapy, and immunotherapy. However, the optimal management approach for recurrent or metastatic ACC is still under investigation, and there is no consensus on the best treatment strategy [30,31,32]. Overall, the outlook for ACC depends on the individual case and requires a personalized approach to treatment and monitoring.

In summary, the treatment and management of subglottotracheal ACC in pediatric patients require a multidisciplinary approach and should be tailored to the individual patient’s needs and circumstances. The goal of treatment is to remove the tumor while preserving as much normal tissue and function as possible and to prevent recurrence and minimize long-term effects of treatment.

## 4. Conclusions

In conclusion, subglottotracheal cystic adenoid carcinoma (ACC) is a rare and challenging disease in pediatric patients. Diagnosis is often delayed due to non-specific symptoms, and a multidisciplinary approach is required for optimal management. Treatment options include surgery, radiotherapy, and chemotherapy, with the choice depending on the stage and location of the tumor. In our case, the patient opted for proton radiotherapy in another specialty center, and after 2 years of follow-up in our center, no side effects of the treatment were observed. Additionally, the patient has no metastases or loco-regional recurrences. However, long-term follow-up is needed to assess potential late effects of radiotherapy. The prognosis of pediatric patients with ACC is variable, with a high risk of relapse and distant metastases, highlighting the need for close monitoring and continued research into new treatment options.

## Data Availability

The data generated in this study may be requested from the corresponding author.
